# Genomic structure and functional trait variation are decoupled across the Atacama–Patagonia arid gradient in the Chilean wineberry

**DOI:** 10.3389/fpls.2025.1741939

**Published:** 2026-02-04

**Authors:** Sebastián Cordero, Gastón O. Carvallo, Tania Coronado, Marcelo R. Rosas, Monique Romeiro-Brito, Lucas C. Majure, Alfredo Saldaña, Heidy M. Villalobos-Barrantes, Pablo C. Guerrero

**Affiliations:** 1 Instituto de Biología, Facultad de Ciencias, Pontificia Universidad Católica de Valparaíso, Valparaíso, Chile; 2 Departamento de Botánica, Facultad de Ciencias Naturales & Oceanográficas, Universidad de Concepción, Concepción, Chile; 3 Institute of Ecology and Biodiversity (IEB), Concepción, Chile; 4 Instituto Forestal, Sede Diaguitas, La Serena, Chile; 5 Department of Natural History, Florida Museum of Natural History, University of Florida, Gainesville, FL, United States; 6 Centro de Investigación en Biología Celular y Molecular, Universidad de Costa Rica, San José, Costa Rica; 7 Escuela de Química, Universidad de Costa Rica, San José, Costa Rica; 8 Millennium Institute Biodiversity of Antarctic and Subantarctic Ecosystems (BASE), Santiago, Chile

**Keywords:** abiotic stress, *Aristotelia chilensis* (maqui), common garden, local adaptation, plant–environment interactions, genomic structure, functional traits, phenotypic plasticity

## Abstract

Local adaptation to aridity is often expected to promote genomic divergence by favoring the integration of drought-tolerance traits. Under this framework, functional trait variation should align with genetic structure; however, empirical evidence for such coupling remains limited, particularly when experimental validation is lacking. We tested this prediction in *Aristotelia chilensis*, a phenotypically variable tree spanning a 1,500-km precipitation gradient (<100 to >1,000 mm year^−1^). We combined nextRAD population genomics, trait–environment modeling, and a common garden drought experiment to assess how climatic and edaphic factors shape genomic structure, drought-related functional traits, reproductive traits, and antioxidant profiles. We identified four genetically distinct clusters that correspond to major biomes across the species’ range—from the Atacama Desert to northern Patagonia—reflecting strong spatial genetic structuring. In contrast, functional traits were largely decoupled from genomic structure and responded independently to environmental variables. Critical photo-inactivation water content (SWC-PhI) showed no credible environmental associations but exhibited significant hierarchical variation among populations and clusters. Specific leaf area (SLA) was strongly influenced by edaphic conditions, decreasing with soil sand content and increasing with soil water-retention capacity, with most variation attributable to population-level differences. Root–shoot biomass ratio also varied hierarchically but was unrelated to climatic or soil predictors. Survival under experimental drought was uniformly low (1.7%) and did not differ among populations or clusters, indicating conserved physiological tolerance across the range. Together, these findings reveal that adaptation to aridity in *A. chilensis* arises from trait-specific, uncoupled responses rather than from an integrated drought-resistance syndrome. The pronounced genomic structure appears more consistent with historical biogeographic processes than with contemporary drought adaptation. These insights underscore the importance of selecting genotypes based on empirical trait performance under water stress—rather than geographic origin—to support climate-resilient fruit production and guide restoration strategies involving *A. chilensis*.

## Introduction

Arid conditions act as a strong selective regime that shapes species distributions and promotes the evolution of drought-tolerance and water-use strategies (Violle et al., 2007; Reich, 2014; Li et al., 2018). These aridity-driven pressures also contribute to the genetic structuring of plant populations (Schierenbeck, 2017). As plants may adopt divergent strategies to cope with water limitation—such as drought avoidance versus drought tolerance—selection is expected to act on suites of traits rather than on single traits in isolation. Accordingly, drought-related traits including leaf morphology, stomatal density, and root architecture (Kosma et al., 2009) often respond to correlational selection (Lowry, 2012; Sexton et al., 2014; Yin et al., 2018; Zhang et al., 2019), leading to coordinated trait shifts (“trait integration”) that underpin functional divergence among locally adapted populations (Izuno et al., 2017; López-Goldar and Agrawal, 2021; Sekely et al., 2024). Understanding the basis of phenotypic integration in response to drought is essential for predicting plant evolutionary trajectories in a changing climate.

Across aridity gradients, plants may adopt divergent strategies—such as drought avoidance versus drought tolerance—that shape the degree of trait integration, the coordinated expression of multiple functional traits (Miao et al., 2025). Such integration often arises from ecological trade-offs or correlational selection, producing characteristic drought-tolerance syndromes (Dayer et al., 2022; Bernardo et al., 2025). For example, under moisture stress, leaf economic traits (e.g., specific leaf area (SLA), leaf N) frequently decouple from hydraulic traits (e.g., vulnerability to cavitation, xylem conductivity), resulting in reduced trait correlations and thus statistical independence across organs or functions (Li et al., 2015). Nonetheless, some systems exhibit the opposite pattern, with strong correlations between hydraulic and structural traits under drought (Medeiros et al., 2025).

Plant responses to water limitation operate across multiple functional levels. Vegetative traits such as leaf morphology, biomass allocation, and specific leaf area often shift as plants adjust carbon investment and tissue construction to enhance water-use efficiency (Kleine et al., 2017; Kapoor et al., 2020). Meanwhile, drought stress frequently impairs photosynthetic performance: water shortage can limit CO_2_ uptake via stomatal closure, destabilize photosystem function, and reduce photochemical efficiency (Chauhan et al., 2023). These physiological disruptions may increase sensitivity to photo-inactivation and limit carbon assimilation, reducing growth under chronic drought.

At the morphological and allocation level, drought often drives increased root–shoot (R:S) biomass ratio or enhanced root investment, at the expense of aboveground growth, as part of a resource-conservation or water-foraging strategy (Ahluwalia and Gupta, 2021). Root architectural changes—including deeper rooting and greater root mass fraction—enable better access to soil moisture and facilitate survival during prolonged water deficits (Sun et al., 2024). At the biochemical level, drought commonly induces the accumulation of secondary metabolites—particularly antioxidants, phenolic compounds, and other reactive-oxygen–scavenging substances—that mitigate oxidative stress associated with dehydration and metabolic disruption (ElSayed et al. 2019). Such biochemical responses contribute to maintaining cell integrity and redox homeostasis under water limitation (Farooq et al., 2015). Because drought often covaries with other environmental drivers—including increased temperature, higher evaporative demand, and elevated irradiance—an integrated view combining physiological thresholds (e.g., photo-inactivation water content), morphological adjustments (SLA, R:S allocation), and biochemical resilience (antioxidants, phenolics) is critical to understand how plants cope across environmental gradients. This multilevel complexity underscores the need for broad, integrative frameworks when investigating trait variation and potential adaptive responses in species distributed across diverse climatic landscapes (Chauhan et al., 2023).

In addition to adaptive processes, neutral evolutionary forces can also structure genomic variation across environmental gradients. Spatial heterogeneity in gene flow, genetic drift, and historical demographic dynamics may generate patterns of population differentiation that mimic or obscure signatures of local adaptation (Slatkin, 1987; Orsini et al., 2013). Climatic refugia, postglacial expansions, and restricted dispersal have been shown to leave strong phylogeographic imprints in many temperate plant species, including lineages distributed along the Andes and coastal ranges of southern South America (Sérsic et al., 2011). Because such neutral processes can confound trait–environment associations, disentangling adaptive divergence requires considering both genomic structure and environmental gradients in a joint analytical framework (Savolainen et al., 2013).

Several studies use correlative approaches to infer trait–environment associations (Butler et al., 2017; Boonman et al., 2021), but experimental assessments are scarce for disentangling causal relationships between water availability and trait variation. Here, we investigate the adaptive basis of ecotypic differentiation in *Aristotelia chilensis* (Molina) Stuntz (Elaeocarpaceae), a Chilean wineberry distributed along a pronounced aridity gradient, from the southern Atacama Desert (<100 mm/year) to northern Patagonia (>1000 mm/year). Its ecological dominance and morphological variability, along with its antioxidant-rich fruits, which hold both cultural and economic significance (Fredes et al., 2012; Schreckinger et al., 2010), make it well suited to assess how genetic structure and trait variability emerge in response to drought. By integrating population genomics, functional trait analysis, and common garden drought experiments, we aim to understand how a drought gradient drives adaptation in this species. Studies integrating ecological, phenotypic, environmental and genomic data are necessary to strengthen inferences about spatial patterns of adaptation (Funk et al., 2012). Specifically, we hypothesize that *A. chilensis* exhibits local adaptation along an aridity gradient, reflected both in pronounced genomic structure and coordinated expression of drought-response traits across geographic regions.

## Materials and methods

### Study areas and plant sampling

**Figure 1:**
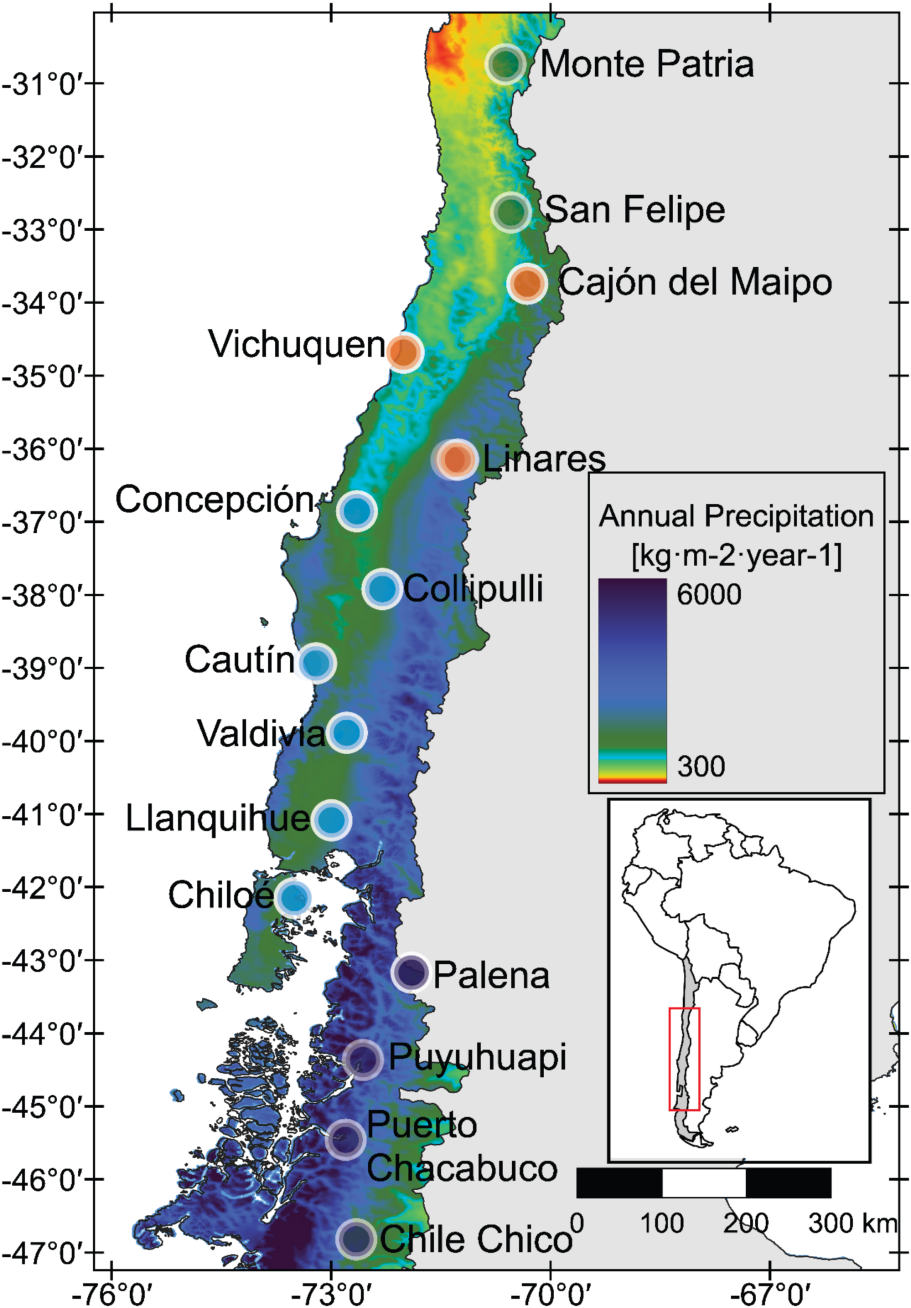
Geographic distribution of *Aristotelia chilensis* populations sampled across their entire latitudinal range in Chile. Colors of each population symbol depict the four genetic clusters identified: North (green), North-central (orange), South-central (light blue), and South (dark blue).

*Aristotelia chilensis*, locally known as maqui, is a wintergreen tree or shrub distributed across Mediterranean and temperate zones of Chile (30–46° S). The species occupies hillsides, forest edges, and riparian ecotones with deep, moist soils, and occurs from coastal lowlands to Andean foothills at elevations up to 2,500 m. Across this range, *A. chilensis* experiences striking climatic heterogeneity, spanning one of the steepest precipitation gradients in southern South America—from hyper-dry Mediterranean sites with <200 mm/year to temperate rainforest conditions exceeding 2,000 mm/year (**Supplementary Figure S1**). This broad hydroclimatic gradient is further shaped by latitudinal changes in seasonality, cloud cover, and evaporative demand, all of which influence plant water balance. To capture this environmental diversity, we sampled 15 populations between December 2019 and February 2020, covering the full latitudinal distribution of the species. We employed a systematic sampling design in which populations were spaced approximately one degree of latitude apart (~110 km; **Figure 1A**), ensuring consistent coverage along the climatic gradient. This design allowed us to encompass strong contrasts in water availability, including a pronounced gradient in potential evapotranspiration, a key indicator of atmospheric water demand that shapes plant physiological performance and adaptation (Fisher et al., 2011).

From each population, between December 2019 and February 2020, we randomly selected 15 adult individuals for molecular analyses—a sample size adequate for capturing within-population genetic variation, which is known to be relatively low in *A. chilensis* (Cona et al., 2023). Individuals were spaced at least 20 m apart to reduce the likelihood of sampling close relatives, and young leaves were collected, flash-frozen in liquid nitrogen, and stored at −80 °C until DNA extraction. To characterize reproductive and antioxidant traits, we sampled 12 of these individuals in most populations (**Table 1**), collecting 10 ripe fruits from each tree for measurements of fruit weight and seed number (1,800 fruits total). Fruits were collected in four field campaigns, from December 2019 to February 2020, starting from the northern area where fruits ripen earlier. For antioxidant analyses, we collected 20 fruits from each of 20 individuals per population (the same 12 individuals plus 8 additional trees); all fruits were immediately frozen in liquid nitrogen. For the common-garden drought experiment, seeds were obtained from these same 20 maternal trees per population. These seeds were germinated under controlled greenhouse conditions near Concepción (36°47′ S, 73°07′ W), a location situated near the midpoint of the species’ geographic distribution. Once seedlings reached transplant size, 20 seedlings per maternal tree (N = 6,000) were established in standardized substrate, of which 15 per maternal tree were assigned to the water-restriction treatment for functional trait and survival analyses.

### DNA extraction, library preparation, and sequencing

Genomic DNA was extracted from the leaves using the DNeasy Plant Mini Kit (QIAGEN), following the manufacturer’s protocol. DNA was extracted until at least 50 ng was obtained and then diluted to a concentration between 0.5 and10 ng/μl, with a maximum of 25 ng/μl. The quality and quantity of extracted DNA were verified using a NanoDrop microvolume UV-Vis spectrophotometer and agarose gels.

Library preparation, sequencing, and genotyping using the nextRAD approach were performed by SNPsaurus LLC (University of Oregon, USA). Genomic DNA was converted into nextRAD genotyping-by-sequencing libraries as in Russello et al. (2015); previously was fragmented with Nextera reagent (Illumina, Inc), which ligates short adapter sequences to the ends of the fragments. The Nextera reaction was scaled for fragmenting 5 ng of genomic DNA, although 5–7 ng of genomic DNA was used for input to compensate for degraded DNA and to increase fragment sizes. Fragmented DNA was then amplified for 26 cycles at 73°C, with one of the primers matching the adapter and extending nine nucleotides into the genomic DNA, with the selective sequence GTGTAGAGC. Thus, we only amplified fragments starting with a sequence that can be hybridized by the selective sequence of the prime. The nextRAD libraries were sequenced on a HiSeq 4000 with two lanes of 150 bp reads (University of Oregon, USA).

### Data filtering and population genetic structure

Genotyping was conducted using custom pipelines developed by SNPsaurus LLC. Raw sequence reads were first processed with *bbduk* from the BBMap package (http://sourceforge.net/projects/bbmap/), which removed adapter sequences, low-quality bases (Phred < 10), and reads shorter than 100 bp. Trimmed reads were then aligned to the *A. chilensis* reference genome (Bastías et al., 2019) using *bbmap* v38.9 with an alignment identity threshold of 0.95, and preliminary variant calling was performed with the same software. As part of the SNPsaurus internal pipeline, alleles with very low population frequency (<3%) were removed to eliminate potential sequencing artifacts. We then applied an additional round of standardized filtering using VCFtools (Danecek et al., 2011) to ensure rigorous SNP selection. We retained only biallelic SNPs, excluded individuals with more than 30% missing data, and removed loci with excessively high read depth (>400) to avoid potential paralogous regions. Finally, we kept variants with a minor allele frequency (MAF) > 0.01. After all filtering steps, the final dataset consisted of 2,023 high-quality SNPs genotyped in 133 individuals (59% of those collected).

We assessed population diversity metrics, including expected (H_e_) and observed heterozygosity (H_o_), inbreeding (F_IS_), and fixation index (F_ST_) for each population, as well as pairwise F_ST_ estimates with confidence intervals based on 1000 bootstrap. We used the package diveRsity (Keenan et al., 2013) implemented in R v4.3.2 (R Core Team, 2024). Population structure and admixture among populations was inferred using three clustering methods. We used the Bayesian clustering algorithm in STRUCTURE v2.3.4 (Pritchard et al., 2000), applying the admixture model with correlated allele frequencies, a burn-in of 1,000,000, and 100,000 Markov chain Monte Carlo (MCMC) replicates. Parameters were calculated for K values ranging from 1 to 10, running 10 iterations and averaging the log probabilities (Ln P) of the data (D) [Ln P(D)]. The most probable K value was evaluated following Evanno et al. (2005). Third, we estimated genetic clustering for geographic patterns using GENELAND v.4.0.6 (Guillot et al., 2005), using the same MCMC number, independent runs, and K such as STRUCTURE. We set the burn-in period to 200 iterations and used an uncorrelated allele frequency model.

### Characterization of reproductive traits

We assessed the variation among populations of three reproductive-related traits: fruit weight, and the number of seeds per fruit. Fruits were collected only at full maturity, identified by their characteristic dark-purple coloration, which ensured consistent developmental stage across populations and minimized variation in fruit weight and seed viability. We collected fruits in four field campaigns, from December 2019 to February 2020, starting from the northern area where fruits ripen earlier. For each tree, we weighed fruits and counted the number of seeds in each fruit, distinguishing viable seeds (with endosperm) from non-viable seeds (without endosperm).

### Antioxidant content and antioxidant activity of fruits

The total polyphenol content was analyzed using the Folin-Ciocalteu reagent, which reacts with polyphenols to produce a blue coloration (Zargoosh et al., 2019). Polyphenol concentration was determined from a calibration curve prepared with gallic acid (3,4,5-trihydroxybenzoic acid) dilutions ranging from 0 to 420 mg/L, with absorbance measured in a spectrophotometer at 750 nm. Each sample was analyzed in triplicate, and the absorbance value per sample was estimated as the mean of the three replicates. The total anthocyanin content was determined using different concentrations of pelargonidin. Samples were incubated with 1% trifluoroacetic acid (TFA) in methanol and refrigerated for 24 hours. Absorbance was measured at 515 nm, and the total anthocyanin content was calculated in terms of chlorinated pelargonidin.

The evaluation of antioxidant capacity was carried out using the ABTS (2,2’-azino-bis(3-ethylbenzothiazoline-6-sulfonic acid)) and DPPH (2,2-diphenyl-1-picrylhydrazyl) radical scavenging assays. For the ABTS assay, extracts were prepared at different concentrations, and the percentage of reduction was calculated. For the DPPH assay, extracts were prepared at different concentrations, and the concentration of extract inhibiting 50% of the DPPH (IC_50_ value) were estimated. Trolox equivalents were determined based on a calibration curve. Antioxidant activity by DPPH radical inhibition was determined using extracts at 50 mg/ml. Samples were diluted to concentrations of 1, 2, 3, 4, and 5 mg/ml. The assay included 200 µl of DPPH solution (20 mg/L) and 100 µl of each sample concentration. After incubating the samples at room temperature for 20 minutes, absorbance was measured at 515 nm. The IC_50_ values were calculated using the polynomial equation in Derive 6.10 Software. Lower IC_50_ values indicate that a smaller concentration of the extract is required to inhibit 50% of the radicals.

### Influence of environmental variables on reproductive and antioxidant-related traits

We selected a set of climatic and edaphic variables associated with aridity to capture the environmental factors shaping *A. chilensis* adaptation across its range, based on previous studies demonstrating their influence on plant physiology (González-Teuber et al., 2018; López-Bucio et al., 2003; Osakabe et al., 2014; Robson et al., 2015; Tanaka et al., 2019). Climatic data included mean annual precipitation (PPT), potential evapotranspiration (PET), and the aridity index (AI = PPT/PET), all obtained from the CHELSA climate database for 1981-2010 (30 arcsec resolution; Karger et al., 2017). We also included ultraviolet-B radiation (UV-B) from the glUV dataset (15 arc-minute resolution; Beckmann et al., 2014).

Edaphic variables included bulk density, total nitrogen, soil organic carbon, coarse fragments, pH, cation exchange capacity, and the proportions of sand, silt and clay, retrieved from the SoilGrids dataset (250 m resolution; Poggio et al., 2021). Additionally, soil water content was obtained from the OpenLandMap Soil Water Content dataset at 33kPa (250 m resolution; Hengl and Gupta, 2019), considering mean values across the top 0.6 m soil depth. Due to high multicollinearity among environmental predictors, we used Lasso regression for variable selection. The final subset included AI, PET, PPT, and UV-B as climatic variables. For edaphic variables were proportion of clay, nitrogen, coarse fragments, cation exchange capacity, and soil organic carbon. All selected variables showed acceptable multicollinearity (VIF < 5).

We employed Bayesian multivariate mixed models to assess the influence of environmental variables independently on antioxidant traits (anthocyanins, phenolics, ABTS, and DPPH) and reproductive traits (fruit weight, total seeds, and viable seeds) using the R package MCMCglmm (Hadfield, 2024). Response variables were z-standardized (zero mean and unit variance). In addition, we incorporated populations and genetic clusters (see genomic section) as a nested random effect with unstructured covariance matrices to account for hierarchical spatial structure. In this way, we assessed the influence of population (lower spatial hierarchy) and genetic cluster (higher spatial hierarchy) on the variance of the studied traits. Bayesian models were run (200,000 iterations and burn-in period of 40,000 iterations) and a thinning interval of 80 to ensure adequate mixing and convergence of the Markov chains.

### Common garden drought tolerance experiment and functional traits characterization

A water-deprivation experiment was conducted to evaluate drought effects on survival and functional traits across populations. Seeds were sown under controlled greenhouse conditions, and once seedlings developed two pairs of true leaves, they were transplanted into standard nursery-grade plastic bags (≈2 L) designed to ensure adequate drainage. Each bag was filled with a homogeneous commercial substrate composed of a peat-based mixture with added perlite and organic soil, a formulation widely used for seedling establishment due to its high aeration and stable water-holding properties. This standardized substrate minimized heterogeneity in soil physical conditions and ensured consistent drying dynamics during the water-restriction treatment. Seedlings were maintained on raised benches inside the greenhouse until the onset of the experiment.

**Figure 2:**
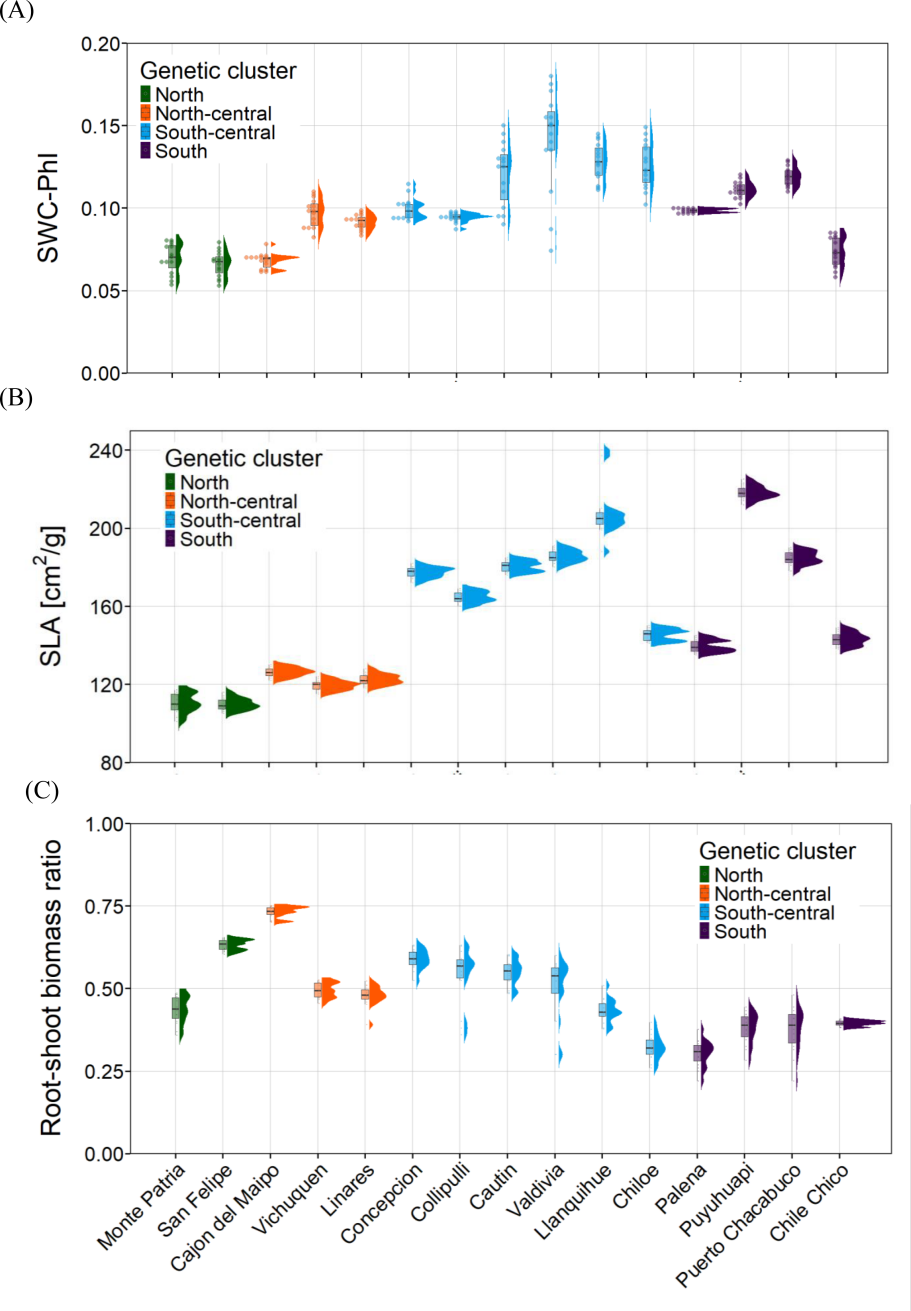
Variation of vegetative functional traits across fifteen *Aristotelia chilensis* populations measured in the drought common-garden experiment on plant seedlings. **(A)** Critical soil water content for photo-inactivation (*SWC-PhI*) of saplings. **(B)** Specific leaf area (SLA) of saplings. **(C)** Root–shoot biomass ratio.

At eight months of age (≈30 cm tall), irrigation was completely ceased to impose drought stress until individuals reached the permanent wilting point (PWP; Rice and Knapp, 2008). Volumetric soil water content (SWC) was monitored at 0–14 cm depth using TMS-3 sensors (TOMST), providing an estimate of water availability in the root zone (Kukal and Irmak, 2023). Soil drying progressed gradually over the two-month experiment (5 August–5 October 2020), and the PWP occurred consistently within an SWC range of 0.07–0.14 m^3^ m^−3^, corresponding to the inflection zone in the drying curve where plants exhibited irreversible loss of turgor (**Figure 2A**).

Determination of the PWP integrated both soil and physiological criteria. For each individual, we monitored maximum photochemical efficiency (Fv/Fm) using a modulated fluorometer (Pocket-PEA, Hansatech Instruments). Permanent wilting was defined as the point at which plants showed (i) sustained decline in Fv/Fm, indicating permanent photo-inactivation (PhI), and (ii) failure to recover turgor or photochemical efficiency after a two-week rewatering test. Across all populations, seedlings reached the PWP after 5–6 weeks of water deprivation, with no significant differences among populations or genetic clusters in the time to reach this threshold. We did not evaluate potential variation associated with plant size or leaf area.

For each sapling, we quantified three drought-related functional traits: (i) critical soil water content for photo-inactivation (SWC-PhI), (ii) specific leaf area (SLA), and (iii) root–shoot biomass ratio. SWC-PhI is a relative measure of stress impacts on PSII, where higher values indicate greater sensitivity (Larcher, 2000; Neuner and Pramsohler, 2006). Photo-inactivation was calculated as PhI = 1 − [(Fv/Fm)d/(Fv/Fm)c], where (Fv/Fm)c corresponds to well-watered controls and (Fv/Fm)d to values measured during drought. SWC and Fv/Fm were monitored every 4–5 days for six weeks until plants reached PWP, and the SWC-PhI threshold was identified graphically following Saldaña et al. (2014).

Functional traits (SLA and R:S ratio) were measured exclusively in well-watered control individuals to ensure representation of baseline, non-stress phenotypes. SLA was quantified from 2–3 leaves per individual using ImageJ (https://imagej.nih.gov/ij/), followed by drying at 70°C for 72 h to obtain leaf dry mass. Root–shoot biomass ratio was determined in 10–15 dried plants per population at the end of the experiment. Plant survival was recorded as the number of individuals alive at the termination of the water-restriction period.

### Effects of genetic structure on survival and physiological traits

We designed a hierarchical model to assess the effects of population (lower level) and genetic clusters (higher level) on survival using data from the common garden experiment, implementing a Cox proportional hazards model. This model analyzes time-to-event data while accounting for censored observations. To explore pairwise differences in survival between populations and clusters, *post-hoc* comparisons were conducted using the Tukey method with Holm correction for multiple testing. Analyses were performed using the package “survival” (Therneau, 2004) in (R Core Team, 2024).

For each physiological trait, we quantified the effects of edaphic and climatic gradients and of genetic structure using independent hierarchical mixed-effects models. Climatic predictors (aridity index, annual precipitation, UV-B) and edaphic predictors (water-retention capacity, percentage sand and clay, total nitrogen, coarse fragments, cation-exchange capacity, and soil organic carbon) were z-standardized prior to analysis to improve convergence and allow direct comparison of effect sizes. We first fitted Gaussian linear mixed-effects models (lme4) for each trait with the full climatic–edaphic predictor set as fixed effects and random intercepts for cluster and population (Trait ~ AI + PPT + UV_B + WaterRetention + PercentSand + PercentClay + TotalNitrogen + CoarseFragments + CationExchangeCapacity + SoilOrganicCarbon + (1|Cluster) + (1|Population)). These models provided initial estimates of fixed effects and partitioned variance among clusters, populations, and residual error. To obtain fully Bayesian estimates of uncertainty and variance components, we then fitted analogous hierarchical models using MCMCglmm, specifying Gaussian response distributions and random effects for cluster and population. We used weakly informative inverse-Gamma–type priors on residual (R) and random-effect (G) variances (R: V = 1, ν = 0.002; G_1_ and G_2_: V = 1, ν = 0.002 for clusters and populations, respectively). Each trait model was run for 200,000 MCMC iterations, with a burn-in of 40,000 and thinning interval of 80, and convergence was assessed by visual inspection of chains, posterior trace plots of fixed effects and variance components, and effective sample sizes.

## Results

### Genetic diversity and population structure

**Figure 3:**
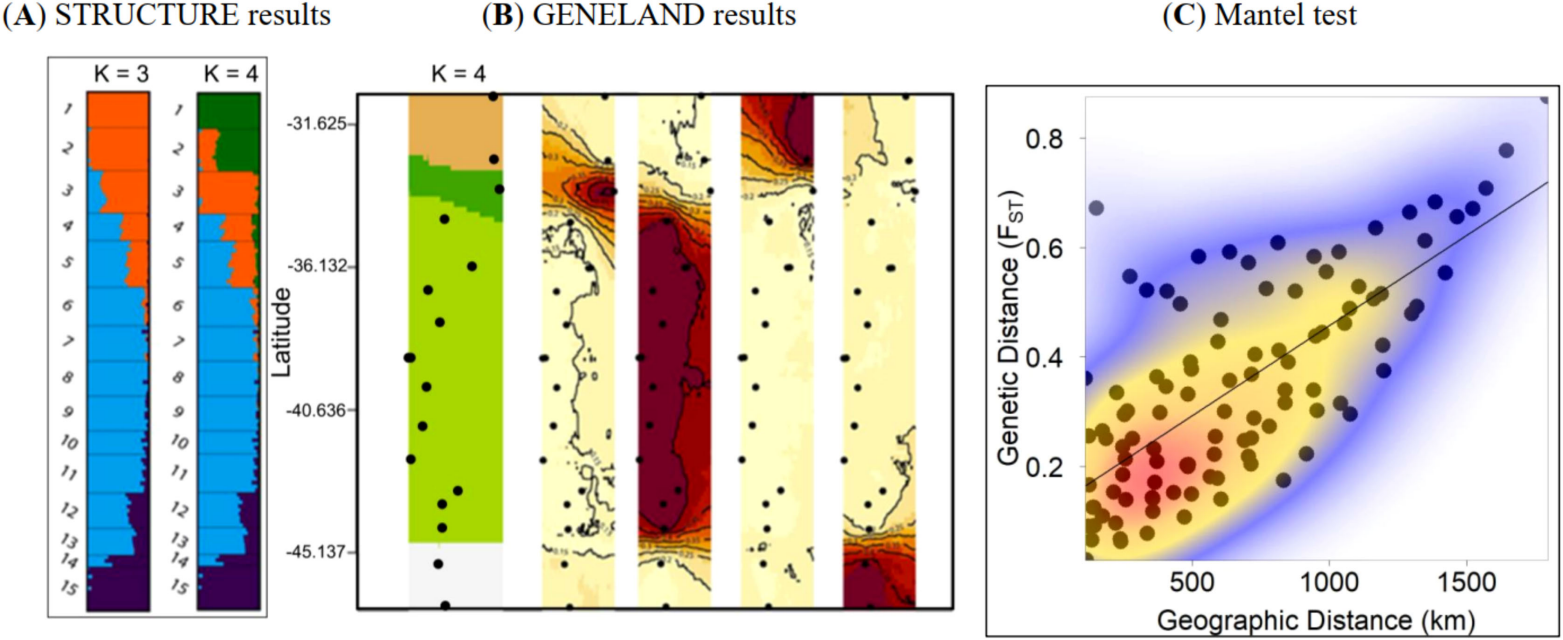
**(A)** Genomic structure of *Aristotelia chilensis* populations inferred using STRUCTURE, showing individual assignment probabilities and revealing four major genetic clusters. **(B)** Spatial genomic structure inferred with GENELAND. The colored spatial bands represent posterior-probability surfaces of membership to each genetic cluster, with every color corresponding to one of the four inferred clusters. These continuous probability fields indicate the likelihood that any given area is dominated by a particular cluster and make spatial transitions in genomic composition visually explicit. **(C)** Relationship between geographic distance (km) and genetic distance (pairwise FST) among populations. The regression line represents the linear fit obtained from the Mantel test, and colors depict kernel density estimation of the pairwise comparisons.

Consistent with the broad latitudinal distribution of *A. chilensis*, which span ca. 1,500 km, we detected four genetic clusters (STRUCTURE analysis, K = 4) and GENELAND—providing convergent evidence for their stability (**Figures 1A**, **3A**): North (include Monte Patria and San Felipe), North-central (Cajon del Maipo, Vichuquen, and Linares), South-central (Concepcion, Collipulli, Cautin, Valdivia, Llanquihue, and Chiloe), and South (Palena, Puyuhuapi, Puerto Chacabuco, and Chile Chico).

The studied populations showed no significant variation in either expected or observed heterozygosity (**Table 1**), and these two metrics were strongly positively correlated (Pearson’s correlation = 0.990; CI_95%_ = 0.970—0.996; t_1,13_ = 25.647; P < 0.001). Observed heterozygosity ranged from 0.02 (Chile Chico) to 0.28 (Vichuquen, Linares and, Cautín), while expected heterozygosity ranged from 0.02 (Chile Chico) to 0.28 (Vichuquen and Linares) (**Table 1**). The inbreeding coefficient (F_IS_) was negative for all populations, indicating an excess of heterozygosity (**Table 1**). Pairwise F_ST_ values indicated shallow genetic differentiation among geographically close populations and among those within the same genetic cluster (**Figures 3C**, **S2**). A Mantel test revealed a significant positive correlation between geographical and genetic distance (r-Mantel = 0.718; P < 0.001; 1000 permutations; **Figure 3C**). For instance, populations from the South-central cluster (Concepción, Collipulli, Cautin, Valdivia, and Llanquihue) showed low pairwise F_ST_ values (0.0284—0.1071), indicating high gene flow (**Supplementary Figure S2**). In contrast, geographically distant populations such as Monte Patria and Chile Chico exhibited one of the highest F_ST_ values, suggesting strong genetic isolation (**Supplementary Figure S2**). Overall, global the F_ST_ and F_IT_ values reached a value of 0.368 (CI_95%_ = 0.355—0.388) and 0.264 (CI_95%_ = 0.242—0.285), respectively.

**Table 1:** Genetic diversity indices and reproductive traits estimated for *Aristotelia chilensis*. Genetic diversity was estimated using 2,023 single nucleotide polymorphisms (SNPs). Populations are ordered from north to south. H_O_: observed heterozygosity; H_e_: expected heterozygosity; F_IS_: inbreeding coefficient; SE: standard error. The Puerto Chacabuco population (N = 3) was retained for completeness; however, its diversity estimates exhibit wide confidence intervals due to the small sample size and should therefore be interpreted with caution.

	Genetic diversity				Reproductive traits			
Populations	Trees successfully genotyped	H_O_	H_e_	F_IS_ (CI_95%_)	Trees sampled	Fruit weight [g] (SE)	Seed number (SE)	Proportion of viable seeds (SE)
Monte Patria	9	0.10	0.08	-0.196 (-0.319—0.054)	12	0.14 (0.01)	3.1 (0.2)	0.92 (0.06)
San Felipe	11	0.21	0.18	-0.179 (-0.268 -0.116)	12	0.11 (0.01)	3.2 (0.2)	0.83 (0.09)
Cajon del Maipo	11	0.25	0.21	-0.229 (-0.315 -0.168)	12	0.15 (0.01)	4.1 (0.1)	0.93 (0.04)
Vichuquen	7	0.28	0.23	-0.207 (-0.333 -0.128)	12	0.13 (0.01)	2.9 (0.2)	0.94 (0.04)
Linares	12	0.28	0.23	-0.177 (-0.244 -0.137)	12	0.13 (0.01)	2.8 (0.2)	0.93 (0.05)
Concepcion	10	0.24	0.2	-0.228 (-0.307 -0.174)	12	0.14 (0.01)	3.0 (0.1)	0.89 (0.05)
Collipulli	9	0.24	0.19	-0.306 (-0.422 -0.166)	12	0.12 (0.01)	3.5 (0.1)	1.0 (0.0)
Cautin	9	0.28	0.21	-0.346 (-0.494 -0.228)	12	0.15 (0.01)	3.6 (0.2)	0.94 (0.03)
Valdivia	9	0.27	0.20	-0.339 (-0.467 -0.234)	12	0.12 (0.01)	3.3 (0.2)	0.92 (0.03)
Llanquihue	6	0.2	0.17	-0.190 (-0.353 -0.080)	4	0.18 (0.01)	4.5 (0.4)	0.95 (0.05)
Chiloe	10	0.19	0.15	-0.263 (-0.399 -0.153)	12	0.17 (0.01)	3.6 (0.2)	0.94 (0.04)
Palena	9	0.16	0.14	-0.181 (-0.291 -0.114)	12	0.15 (0.01)	3.4 (0.2)	0.98 (0.02)
Puyuhuapi	7	0.18	0.15	-0.200 (-0.389 -0.058)	12	0.15 (0.01)	2.9 (0.2)	0.88 (0.06)
Puerto Chacabuco	3	0.05	0.06	0.053 (-0.375—0.650)	12	0.14 (0.01)	3.4 (0.2)	0.98 (0.02)
Chile Chico	11	0.02	0.02	-0.203 (-0.310—0.083)	5	0.12 (0.01)	3.5 (0.3)	0.83 (0.13)
Global	133			-0.163 (-0.199 -0.139)				

### Reproductive trait variation

Reproductive traits showed similar values among studied populations (**Table 1**) with significant effects of edaphic variables on these. Specifically, fruit weight averaged 0.14 g (coefficient of variation; CV = 21%), with 3.5 seeds per fruit (CV = 19,7%), and a mean viability of 93% (CV = 17.2%). We observed that fruit weight, seed number, and the number of viable seeds were statistically associated with soil water retention (β = 0.705, pMCMC = 0.036) and soil organic carbon (β = 0.188, pMCMC = 0.049).

### Antioxidant activity patterns and their relationship with environmental variables

We observed lower variation in antioxidant content (coefficient of variation; CV_Polyphenols_ = 35.4%; CV_Anthocyanin_ = 57.7%) than antioxidant activity (CV_ABTS_ = 52.9%; CV_DPPH_ = 267%) (**Supplementary Table S1**). Antioxidant production was modulated by climatic factors, with significant effect of spatial hierarchical structure at the population and cluster levels. Specifically, PET showed a strong positive association with antioxidant content (β = 0.552, pMCMC = 0.005).

### Common garden experiment: survival and functional traits

After 56 days of drought, only 1.7% of the 3,321 saplings remained alive (**Supplementary Figure S1**). Neither population nor genetic cluster exhibited statistically significant effects on survival in the Cox proportional hazards model (P = 0.600). Hazard ratios indicated only non-significant tendencies: for example, the North cluster showed a slightly higher hazard relative to the North-central cluster (HR = 1.138; CI95% = 0.566–2.289; P = 0.717), whereas the South cluster showed a modestly lower hazard than the South-central cluster (HR = 0.765; CI95% = 0.420–1.393; P = 0.381). At the population level, hazard ratios ranged from 0.799 to 0.814, with none differing significantly from 1 (Wald test: concordance = 0.558; P = 0.900).

Bayesian mixed models (MCMCglmm) revealed that the three functional traits—critical photo-inactivation water content (SWC-PhI), SLA, and R:S biomass ratio—showed substantial hierarchical structure, with most variation attributable to population identity and, to a lesser extent, genetic cluster (**Tables 2**, **S2**; **Figure 2**). In all three traits, both population- and cluster-level variances had posterior distributions whose 95% credible intervals excluded zero, confirming significant hierarchical structuring. For SWC-PhI, none of the environmental predictors exhibited credible effects: all slope estimates had wide 95% credible intervals that overlapped zero (pMCMC > 0.4). Nonetheless, both population (Var = 0.00062, CI95% = 0.00012–0.00137) and genetic cluster (Var = 0.0036, CI95% = 0.00013–0.01198) contributed significantly to SWC-PhI variation, consistent with strong spatial genetic structure. For SLA, the Bayesian model identified clear environmental associations. SLA increased with soil water-retention capacity (β = 10.41, CI95% = 0.48–18.93, pMCMC = 0.027) and decreased with sand proportion (β = −17.38, CI95% = −28.16 to −7.98, pMCMC = 0.001). Precipitation (PPT) showed a positive but non-credible tendency (β = 5.12, CI95% = −0.36–10.52, pMCMC = 0.074). SLA exhibited strong population-level variation (Var = 850.8, CI95% = 224.9–1723) and weaker but non-zero cluster-level variance (Var = 172, CI95% = 0.0002–649.8). For the R:S biomass ratio, none of the environmental variables displayed credible effects (all CI95% overlapped zero). Trait variation was nonetheless structured at both hierarchical levels: population (Var = 0.0092, CI95% = 0.0019–0.0213) and cluster (Var = 0.0163, CI95% = 0.00024–0.066) contributed to observed differences among individuals.

**Table 2:** Summary of Bayesian hierarchical models estimating the effects of climatic and edaphic predictors on three functional traits of *Aristotelia chilensis*: critical photo-inactivation water content (SWC-PhI), specific leaf area (SLA), and root–shoot biomass ratio (R:S). Posterior means (β), 95% credible intervals (CI95%), and pMCMC values are reported. Bold values indicate CI95% that exclude zero, representing statistically credible contributions of environmental predictors to trait variation.

Environmental variable	SWC-PhI β	SWC-PhI CI95%	pMCMC	SLA β	SLA CI95%	pMCMC	R:S β	R:S CI95%	pMCMC
Aridity Index (AI)	−0.0000128	−0.01887 to 0.01717	0.999	0.82583	−16.33060 to 17.10298	0.911	−0.0012671	−0.0728981 to 0.0659607	0.951
Mean annual precipitation (PPT)	0.001903	−0.007737 to 0.01194	0.715	5.12213	−0.36122 to 10.51936	0.074	0.0014177	−0.0359547 to 0.0394506	0.946
UV-B	−0.009356	−0.05016 to 0.03450	0.676	−3.98468	−27.93358 to 20.98169	0.729	−0.0345209	−0.1725079 to 0.1206288	0.648
Water retention capacity	−0.001910	−0.02129 to 0.01636	0.851	10.41120	0.47909 to 18.93029	0.027	−0.0053863	−0.0836853 to 0.0704007	0.874
Percent sand	−0.0003945	−0.01874 to 0.01843	0.963	−17.37774	−28.16448 to −7.97669	0.001	0.0339657	−0.0388193 to 0.1057366	0.342
Percent clay	0.002100	−0.02097 to 0.02530	0.876	−9.25613	−23.29407 to 4.35392	0.178	0.0443571	−0.0378154 to 0.1311343	0.304
Total nitrogen	0.012910	−0.02012 to 0.04282	0.385	−0.05369	−18.23668 to 18.54004	0.987	−0.0855401	−0.2009495 to 0.0326486	0.156
Coarse fragments	0.0005073	−0.01992 to 0.02111	0.974	4.24013	−5.51576 to 13.41129	0.398	0.0330923	−0.0454254 to 0.1162144	0.420
Cation exchange capacity	−0.0008275	−0.02066 to 0.01705	0.943	4.29338	−5.78416 to 12.70275	0.364	−0.0009707	−0.0789340 to 0.0661705	0.981
Soil organic carbon	0.0000607	−0.003066 to 0.003154	0.985	0.11989	−1.14781 to 1.33890	0.851	−0.0033376	−0.0152637 to 0.0088614	0.613

## Discussion

Our genomic analysis of *A. chilensis* revealed four distinct genetic clusters across its distributional range, indicating a pronounced spatial genetic structure likely shaped by the joint influence of historical processes and contemporary environmental heterogeneity, although we did not formally test for adaptive genomic divergence. While these clusters broadly correspond to latitudinal regions (North, North-central, South-central, and South), the observed patterns cannot be explained solely by isolation by distance. Notably, the strong genetic differentiation detected across transitional zones (e.g., between Linares and Concepción) suggests that abrupt environmental boundaries may contribute to structuring gene flow. Similar patterns have been reported for other Chilean temperate trees distributed along extensive latitudinal gradients (Mathiasen and Premoli, 2010; Premoli et al., 2012). The position of the major break between North-central and South-central clusters aligns with a well-established biogeographic discontinuity near 37°S, associated with Pleistocene refugia (Sérsic et al., 2011). Although our study did not perform demographic or coalescent analyses, such correspondence is consistent with historical climatic legacies interacting with present-day environments to generate the observed spatial genomic mosaic.

Trait variation in *A. chilensis* shows no correspondence with phylogeographic structure, indicating that phenotypic divergence is largely independent of postglacial lineage history. This contrasts with expectations derived from genetic structure alone. While previous work (Cona et al., 2023) suggests that Patagonian populations represent recent recolonization from northern refugia, our results show that drought-related functional traits vary across the gradient but do not align with genomic clusters. Taken together, these findings suggest that the spatial patterning of drought-adaptive traits reflects recent and geographically localized adjustments to climatic gradients, rather than the persistence of ancestral trait differences. The absence of integrated trait syndromes across clusters, combined with clear environmental effects on specific traits, supports substantial phenotypic plasticity in this species. Such plasticity may facilitate persistence across steep climatic gradients and buffer populations from ongoing climatic drying (Salazar et al., 2024).

The analysis of reproductive traits and antioxidant production in *A. chilensis* reveals complex spatial structuring and strong associations with environmental variables, particularly edaphic factors for reproduction and climatic variables for antioxidant production. The positive covariation among fruit weight, seed production, and soil properties such as water retention and organic carbon suggests resource-mediated constraints in reproductive allocation (Obeso, 2002; Stearns, 2000). Secondary metabolite production showed strong positive associations with potential evapotranspiration, consistent with their role in mitigating abiotic stress (Nakabayashi and Saito, 2015). The weak correlation between antioxidant production and activity suggests independent regulation of distinct antioxidant pathways (Blokhina et al., 2003), highlighting the complexity of physiological responses to the environment.

The common-garden drought experiment revealed no significant differences in survival among populations or genetic clusters, despite substantial genomic structure. Although hazard ratios indicated mild tendencies at the cluster level, these were not statistically supported. The overall homogeneity in survival suggests that core drought-tolerance mechanisms may be evolutionarily conserved across the species’ range. Several mechanisms could contribute to this pattern, including: (i) complex, polygenic control of drought tolerance not captured by genome-wide neutral markers; (ii) genetic differentiation arising primarily from environmental factors unrelated to drought; and (iii) substantial phenotypic plasticity buffering population-level differences (Valladares et al., 2014). Furthermore, the severity of the drought treatment (98% mortality) may have masked subtler among-population variation in tolerance.

Functional traits also varied independently, showing weak covariation. As a pioneer species adapted to forest edges, *A. chilensis* relies on high fecundity, rapid establishment, and broad ecological tolerance rather than tightly coordinated drought-response syndromes. The independence among functional traits aligns with the “entangled phenotypes” framework (Díaz, 2025), implying distinct, domain-specific axes of trait variation shaped by heterogeneous selective pressures. Such flexible trait combinations may allow populations to respond to diverse local environments rather than conforming to a unified drought-resistance strategy.

Critical photo-inactivation water content (SWC-PhI) showed no credible environmental associations but significant hierarchical variance at both population and cluster levels. This suggests a complex evolutionary signature, possibly combining historical climatic influences with phylogenetically conserved aspects of photosynthetic physiology (Savage and Cavender-Bares, 2012; Brodribb et al., 2020). The multilevel structure of this trait illustrates how photosynthetic drought tolerance emerges from interactions between broad climatic regimes and fine-scale environmental variation (Flexas et al., 2021). In contrast, specific leaf area (SLA) showed clear environmental associations, primarily with edaphic factors: SLA decreased with increasing sand content and increased with soil water-retention capacity, indicating that soil moisture plays a central role in shaping leaf morphology (Lambers et al., 2008; Maire et al., 2015). A positive but non-credible association with precipitation further supports this water-availability pattern. Although population-level variance was large, uncertainty at the cluster level suggests that SLA is mainly driven by local soil conditions rather than macroscale climatic gradients (Reich, 2014). Root–shoot biomass ratio showed no credible environmental associations. This was unexpected given the well-documented plasticity of allocation under water stress (Poorter et al., 2012; Eziz et al., 2017). The significant hierarchical structure, with larger variance at the cluster than population level, is consistent with developmental or phylogenetic constraints (Schall et al., 2018), suggesting that biomass partitioning in this long-lived woody species is relatively stable across environmental gradients (Petit and Hampe, 2006).

Our study provides a broad and integrative view of genomic and phenotypic variation in *A. chilensis*, but several limitations warrant consideration. The SNP density obtained through nextRAD, although sufficient for reconstructing broad-scale population structure, limits detection of finer genomic signals such as selection outliers or genotype–environment associations. Trait–environment effect sizes, while statistically supported for some traits, were modest, restricting the strength of inferences regarding adaptive significance. The extreme severity of the drought treatment reduces our ability to detect more subtle population-level variation in physiological tolerance. Finally, sampling a single generation of a long-lived woody species precludes evaluation of intergenerational shifts in genetic or phenotypic structure.

Overall, our findings indicate that aridity responses in *A. chilensis* arise from decoupled, trait-specific adjustments rather than integrated drought-resistance syndromes. Despite pronounced genomic structure, functional trait divergence was largely independent of genetic clusters. Only specific traits—most notably SLA—showed clear environmental associations, particularly with edaphic variables. This decoupling underscores the complexity of adaptation under heterogeneous selective pressures and highlights the predominant role of environmental filtering in shaping phenotypic expression. These insights have practical implications: selecting planting material based on phenotypic performance under target conditions, rather than geographic origin, may better support climate-resilient cultivation and restoration.

## Data Availability

The data presented in this study are deposited in the Zenodo repository under accession number 17832070 (DOI: 10.5281/zenodo.17832070).
