# Supplementary material for: Genomic structure and functional trait variation are decoupled across the Atacama–Patagonia arid gradient in the Chilean wineberry
Source: Front Plant Sci. 2026 Feb 4;16:1741939. doi: 10.3389/fpls.2025.1741939 (PMC12913428; doi:10.3389/fpls.2025.1741939)
Supplement: Supplementary file 1 [file DataSheet1.docx]

**SUPPLEMENTARY MATERIAL**

**Table S1.** Antioxidant content and activity across populations of *A. chilensis.* For each population, we studied 20 fruits per tree. We reported the mean and standard error (in parenthesis) per population.

|  | Antioxidant content | | Antioxidant activity | |
| --- | --- | --- | --- | --- |
| Populations | Polyphenols | Anthocyanin | ABTS | DPPH |
| Monte Patria | 4.52 (0.19) | 60 (9) | 7.7 (0.7) | 2.3 (0.2) |
| San Felipe | 4.88 (0.15) | 82 (10) | 8.2 (1.2) | 3.9 (1.2) |
| Cajon del Maipo | 3.81 (0.17) | 127 (3) | 2.8 (0.8) | 0.0 (2.3) |
| Vichuquen | 5.77 (0.33) | 93 (12) | 7.0 (0.8) | 1.0 (0.1) |
| Linares | 4.99 (0.29) | 73 (12) | 8.6 (0.9) | 2.4 (0.4) |
| Concepción | 5.25 (0.79) | 190 (13) | 7.3 (0.3) | 3.7 (1.1) |
| Collipulli | 4.35 (0.36) | 192 (16) | 2.9 (0.3) | 5.6 (0.8) |
| Cautin | 5.97 (0.28) | 209 )10) | 7.6 (1.4) | 2.0 (0.3) |
| Valdivia | 5.71 (0.35) | 243 (15) | 3.8 (0.3) | 2.0 (0.5) |
| Llanquihue | 4.58 (0.15) | 98 (13) | 5.2 (0.7) | 1.3 (0.4) |
| Chiloe | 4.44 (0.18) | 56 (11) | 4.7 (0.4) | 1.3 (0.1) |
| Palena | 5.71 (0.61) | 191 (13) | 5.5 (0.1) | -3.7 (2.1) |
| Puyuhuapi | 5.89 (0.98) | 204 (15) | 7.9 (0.4) | -1.1 (0.9) |
| Puerto Chacabuco | 5.50 (0.12) | 249 (6) | 6.6 (0.5) | 3.3 (0.2) |
| Chile Chico | 5.51 (0.06) | 130 (17) | 4.4 (0.4) | 1.5 (0.5) |

**Table S2.** Assessment of covariation (Bayesian multivariate mixed models) among functional traits in *A. chilensis* across genetic clusters. β: covariation coeficient; CI_95%_: 95% credible intervals.

|  | Critical photo-inactivation water content (SWC-PhI) | SLA |
| --- | --- | --- |
| Specific leaf area (SLA) | β = 1.66  CI_95%_ = -122.9–111.7 |  |
| Root: shoot biomass | β= -0.03  CI_95%_ = -2.77–2.62 | β= 0.14  CI_95%_ = -100.90–151.51 |

**
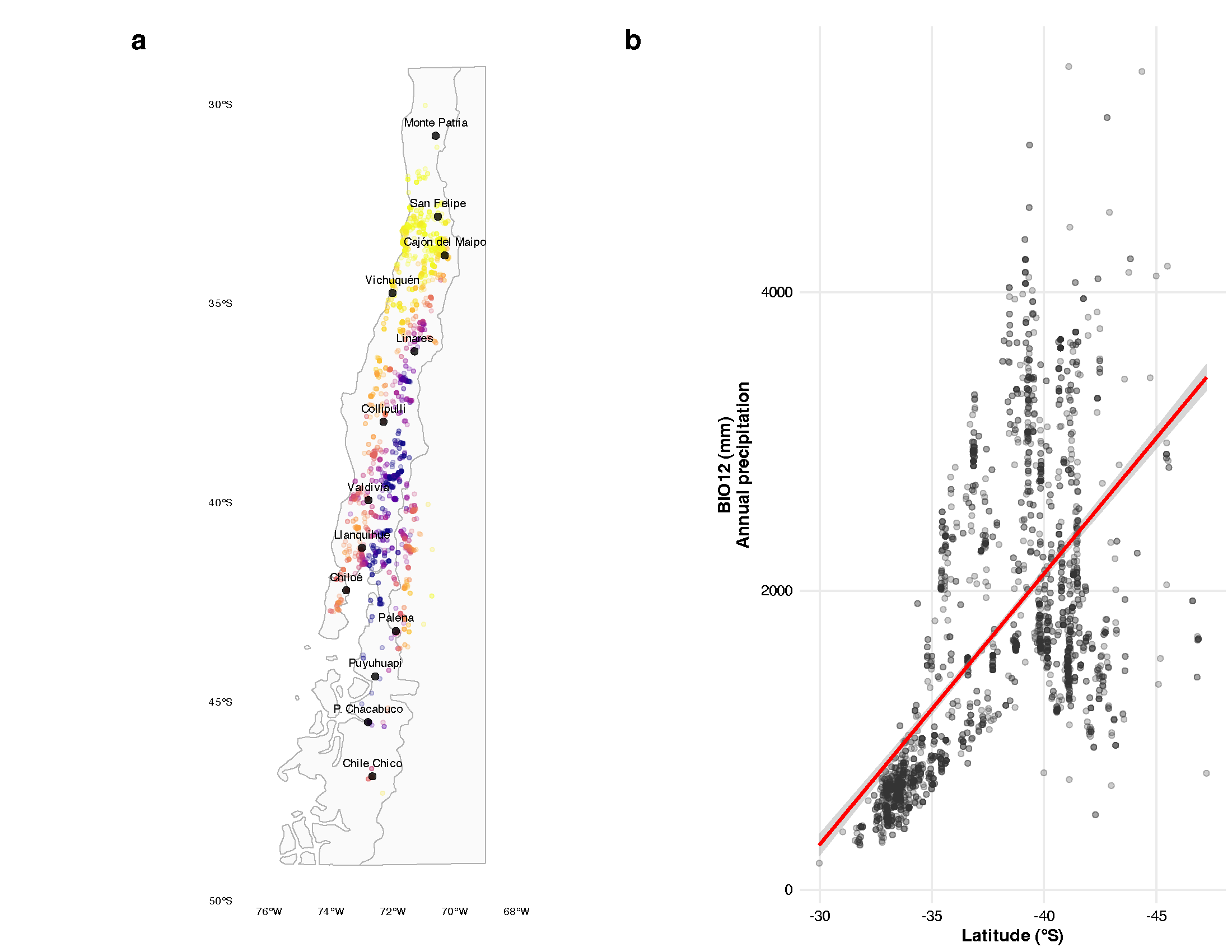
**

**Figure S1.** Spatial distribution and climatic context of Aristotelia chilensis in Chile. **(a)** Occurrence records from GBIF (DOI: 10.15468/dl.tygatx) and iNaturalist, retained after coordinate normalization, duplicate removal, and spatial outlier filtering (**n = 3,359**), shown as semi-transparent points colored by CHELSA v1.2 BIO12. Black dots filled symbols indicate the geographic centroids of the populations included in the genomic sampling. **(b)** Relationship between annual precipitation (BIO12) and latitude for all retained occurrences, with points representing individual records and the red line showing the fitted linear model (±95% CI). A strong increase in precipitation toward higher latitudes is evident, supported by a significant negative correlation (Pearson’s r = –0.618; t = –45.56; df = 3357; p < 0.0001).

**
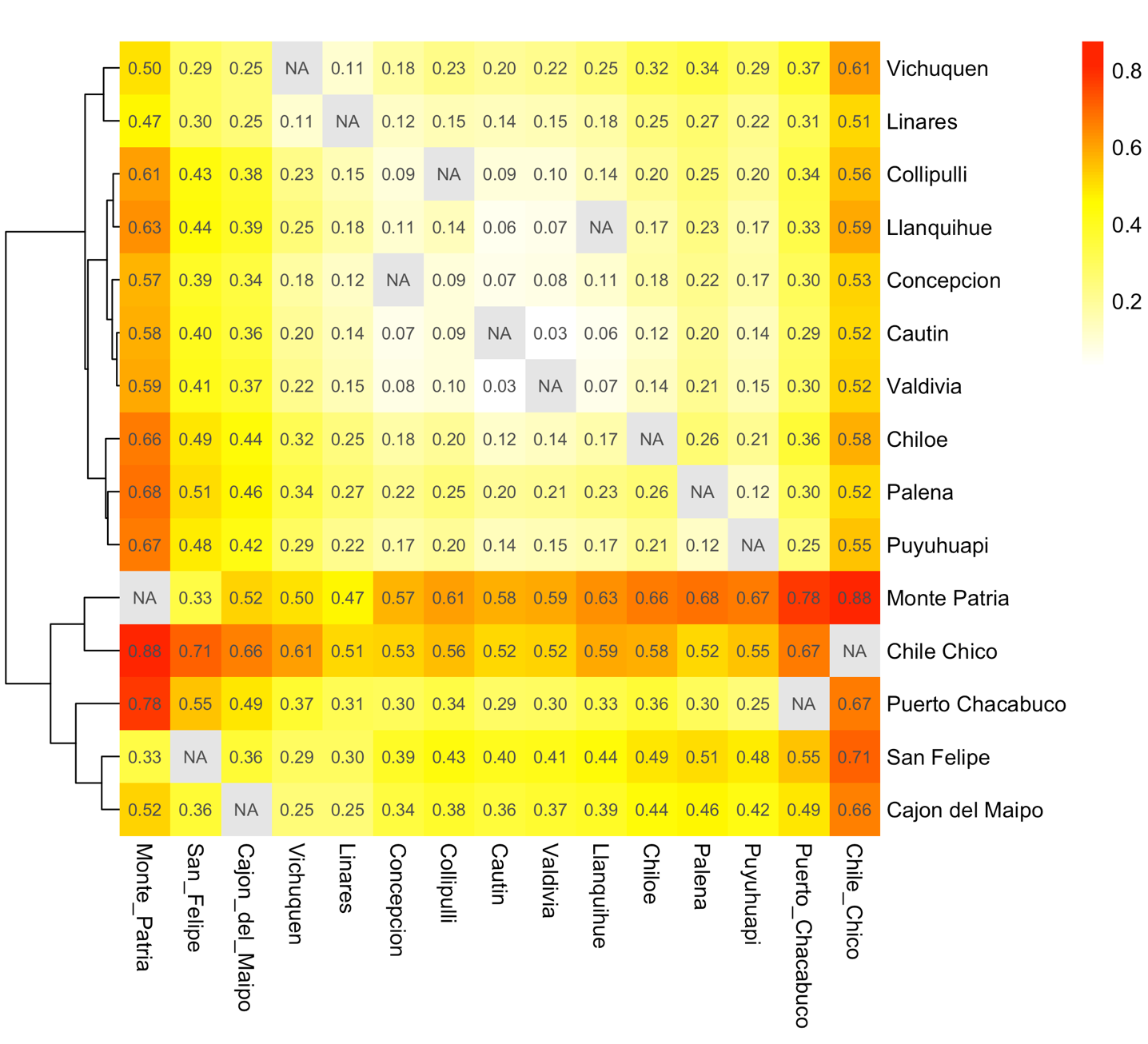
**

**Figure S2. Heatmap of pairwise genetic differentiation (FST) among 15 populations of Aristotelia chilensis.** The matrix displays pairwise FST estimates calculated from 2,023 SNPs, with darker colors indicating higher genetic differentiation and lighter colors indicating lower differentiation among population pairs. Rows and columns are ordered according to hierarchical clustering.


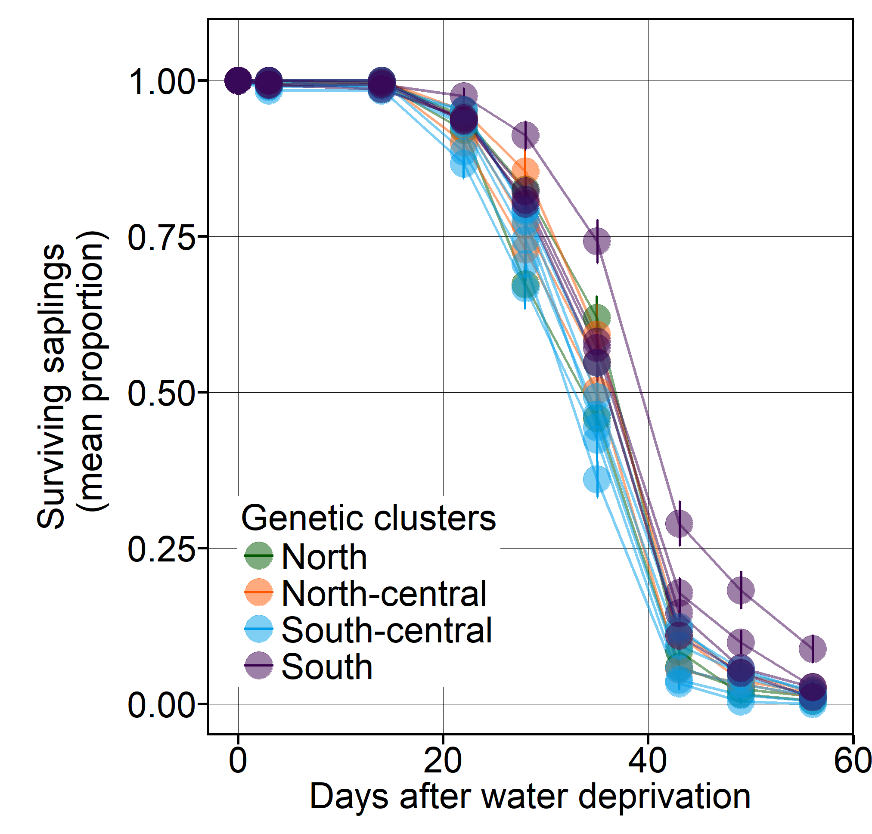


**Figure S3.** Variation of sapling survival over time (days after water deprivation) under greenhouse conditions across fifteen *Aristotelia chilensis* populations (mean ± SE).

**
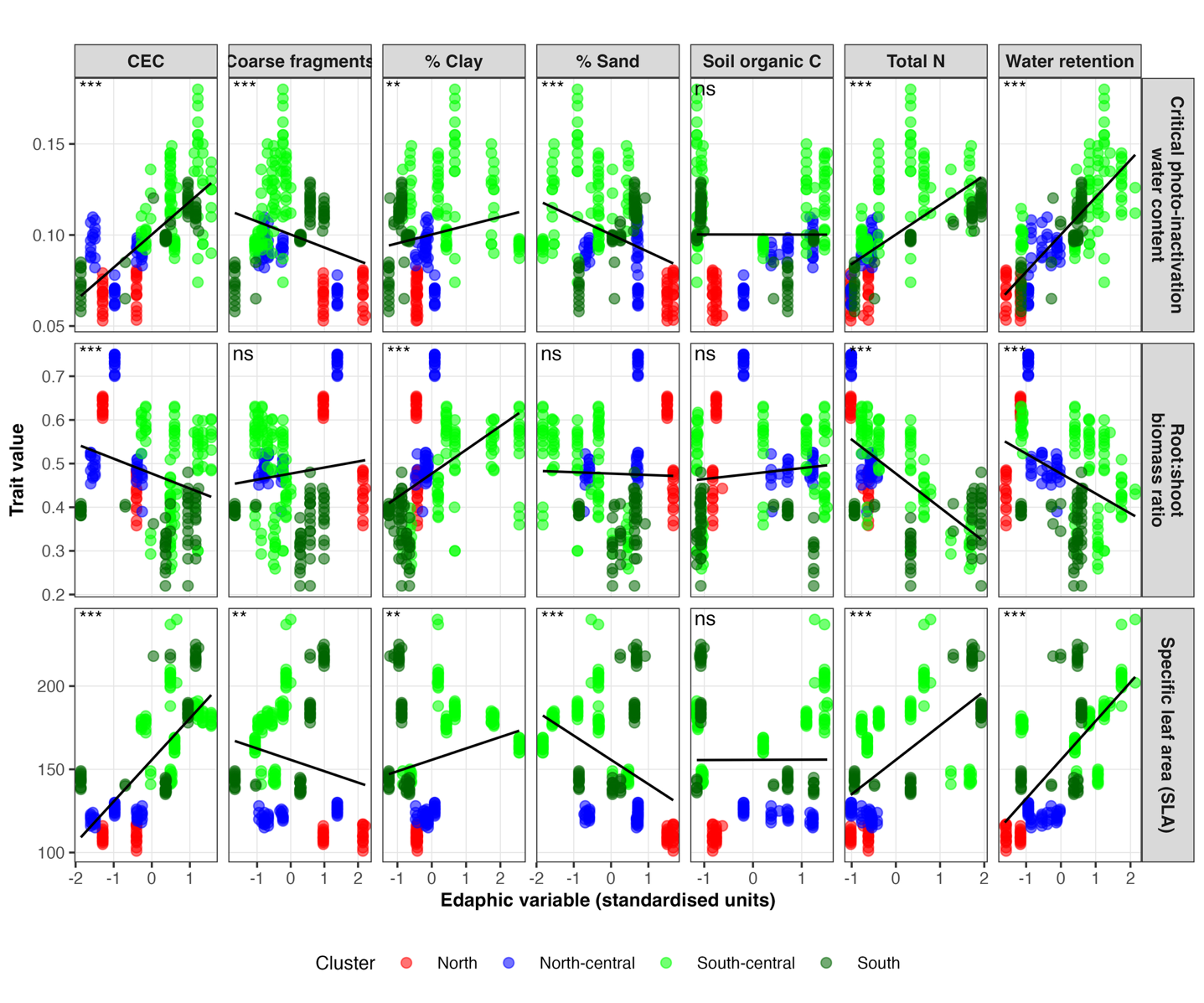
**

**Figure S4.** Relationships between functional traits and edaphic variables across populations of *Aristotelia chilensis*. Each point represents one population mean, colored according to its geographic cluster (“North”, “North-central”, “South-central”, “South”). Edaphic predictors were standardized (z-scores). For visualization, black lines depict ordinary least-squares regressions fitted across all populations for each Trait × Soil Variable combination. The significance of each slope is indicated in the upper-left corner of each panel (*p* < 0.05; **p** < 0.01; ***p*** < 0.001; ns = not significant). Traits include critical photo-inactivation water content, specific leaf area (SLA), and root:shoot biomass ratio. Edaphic variables include water retention capacity, sand and clay content, total nitrogen, coarse fragments, cation exchange capacity (CEC), and soil organic carbon.
